# XII International Mycological Congress: report of Congress action on nomenclature proposals relating to fungi

**DOI:** 10.1186/s43008-024-00169-2

**Published:** 2024-11-21

**Authors:** Tom W. May, Konstanze Bensch, Johannes Z. Groenewald, Jos Houbraken, Amy Y. Rossman

**Affiliations:** 1 Royal Botanic Gardens Victoria, Birdwood Avenue, Melbourne, VIC 3004 Australia; 2 Westerdijk Fungal Biodiversity Institute, Uppsalalaan 8, 3584 CT Utrecht, The Netherlands; 3 Department of Botany & Plant Pathology, Oregon State University, Corvallis, OR 97333 USA; 4 Fungal Nomenclature Bureau, XII International Mycological Congress, Maastricht, The Netherlands

**Keywords:** *Code*, Fungal Nomenclature Bureau, Fungal Nomenclature Session, ICNafp, Nomenclature Committee for Fungi, Special-purpose Committee

## Abstract

Procedures, appointments and outcomes of the Fungal Nomenclature Session (FNS) of the XII International Mycological Congress (IMC12) are summarized, including the composition of the Fungal Nomenclature Bureau and the Nominating Committee of the IMC. Between 124 and 322 mycologists attended the three sessions of the FNS, at which formal proposals to amend *Chapter F* of the *International Code of Nomenclature for algae, fungi, and plants* were debated. The 15 proposals considered included eight "from the floor", five of which were withdrawn prior to the FNS. One of the withdrawn proposals was directed to the Editorial Committee for Fungi, in relation to adding examples of best practice when citing living cultures as types. Among the seven proposals published in *Taxon*, one proposal with a high “no” vote in the *Guiding Vote* was not re-introduced. Discussion on one proposal led to the authorization of a Special-purpose Committees on “Genomes as Types for Fungi”. For the eight proposals that were put to a vote, two proposals were rejected and six proposals were accepted. The accepted proposals: (1) clarified that a proposal to conserve a name with a conserved type does not require citation of a typification identifier; (2) clarified procedures to protect and reject names of fungi; (3) removed the need to list synonyms of protected names in the *Code* appendices; (4) clarified that an earlier homonym of a sanctioned name remains unavailable if the sanctioned name is rejected outright; (5) recommended that culture collections or biological resource centres where cultures are lodged should be “public”; and (6) recommended that when original type cultures are lost, neotypification should utilize the progeny of ex-type cultures. Appointments made by the FNS included the Secretary of the Fungal Nomenclature Bureau for IMC13, the officers and members of the Editorial Committee for Fungi, and the officers and members of the Permanent Nomenclature Committee for Fungi. Decisions and appointments of the FNS were ratified in a resolution accepted by the plenary session of the Congress.

## INTRODUCTION

Provisions of the *International Code of Nomenclature for algae, fungi, and plants* (ICNafp; Turland et al. 2018) that deal solely with names of organisms treated as fungi are included in “*Chapter F*”. Proposed amendments to *Chapter F* are dealt with by the Fungal Nomenclature Session of an International Mycological Congress (IMC) rather than the Nomenclature Section of an International Botanical Congress (IBC). The timetable and procedures for introducing proposals to amend *Chapter F* were outlined by May (2020), with an alternation to the closing date to 31 December 2023 due to the postponement of IMC12 to 2024 (May and Hawksworth 2024); proposals were published (May and Hawksworth 2024); a *Synopsis* of these proposals was presented (May and Bensch 2024); and a pre-Congress *Guiding Vote* on the proposals was held (May and Miller 2024).

The Fungal Nomenclature Session (FNS) of the 12th International Mycological Congress (IMC12) took place on Thursday 15 August 2024 in Auditorium 1 of the MECC Maastricht conference centre, Maastricht, The Netherlands. The FNS was opened by Pedro Crous, Chair of the IMC12 Organizing Committee, who welcomed delegates to the Session.

Any person registered for that day of the Congress was entitled to attend the FNS and vote. There were three sessions: the first and last were included in the Congress program as symposia (Nomenclature A and Nomenclature B) and there was an additional session scheduled during the lunch break, specifically to allow debate on the proposals concerning DNA sequences as types for fungi. The persons attending were counted for each session, but no sign-in sheet was used to keep track of who attended. A special symposium was held on Monday 12 August 2024 to introduce the concept of DNA sequences as types (Symposium 11: DNA sequences as type equivalent—where to next?) to set the stage for voting on the two proposals regarding this topic during the lunchtime session of the FNS on Thursday.

**Fig. 1:**
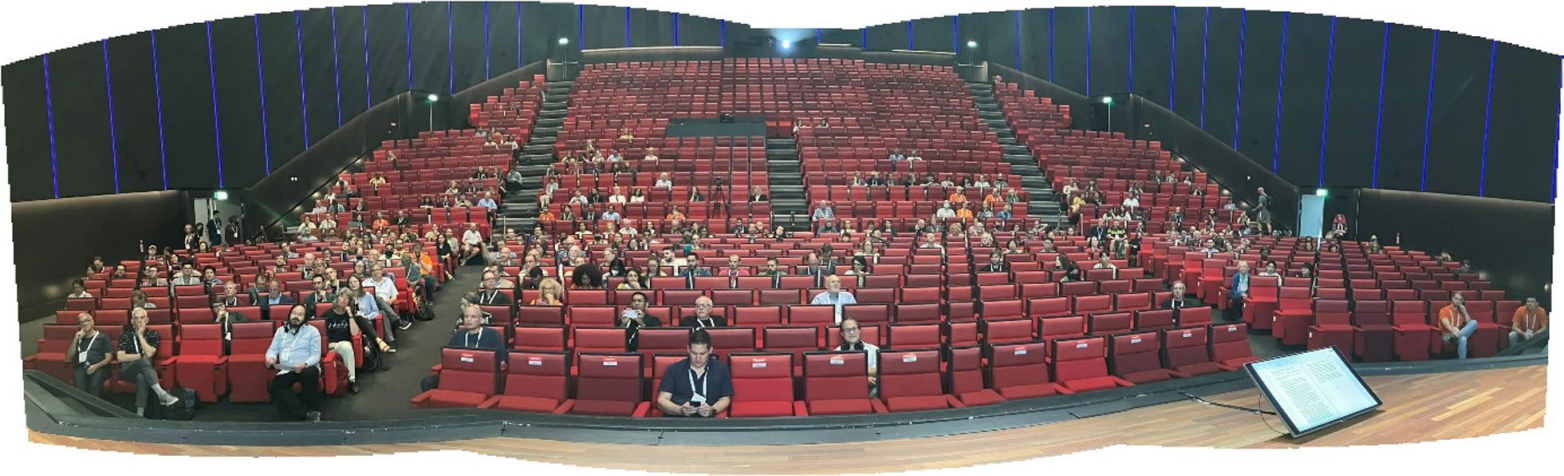
Participants in the Fungal Nomenclature Session of IMC12. Photo: Nicholas Turland

The numbers attending the FNS were: Session 1 (Nomenclature A): 174, Session 2 (Vote on DNA sequences as types proposals): 322 (Fig. 1), and Session 3 (Nomenclature B): 124. In comparison, a total of 149 people attended the FNS at IMC11 in Puerto Rico.

## FUNGAL NOMENCLATURE SESSION PROCEDURES AND APPOINTMENTS

**Fig. 2:**
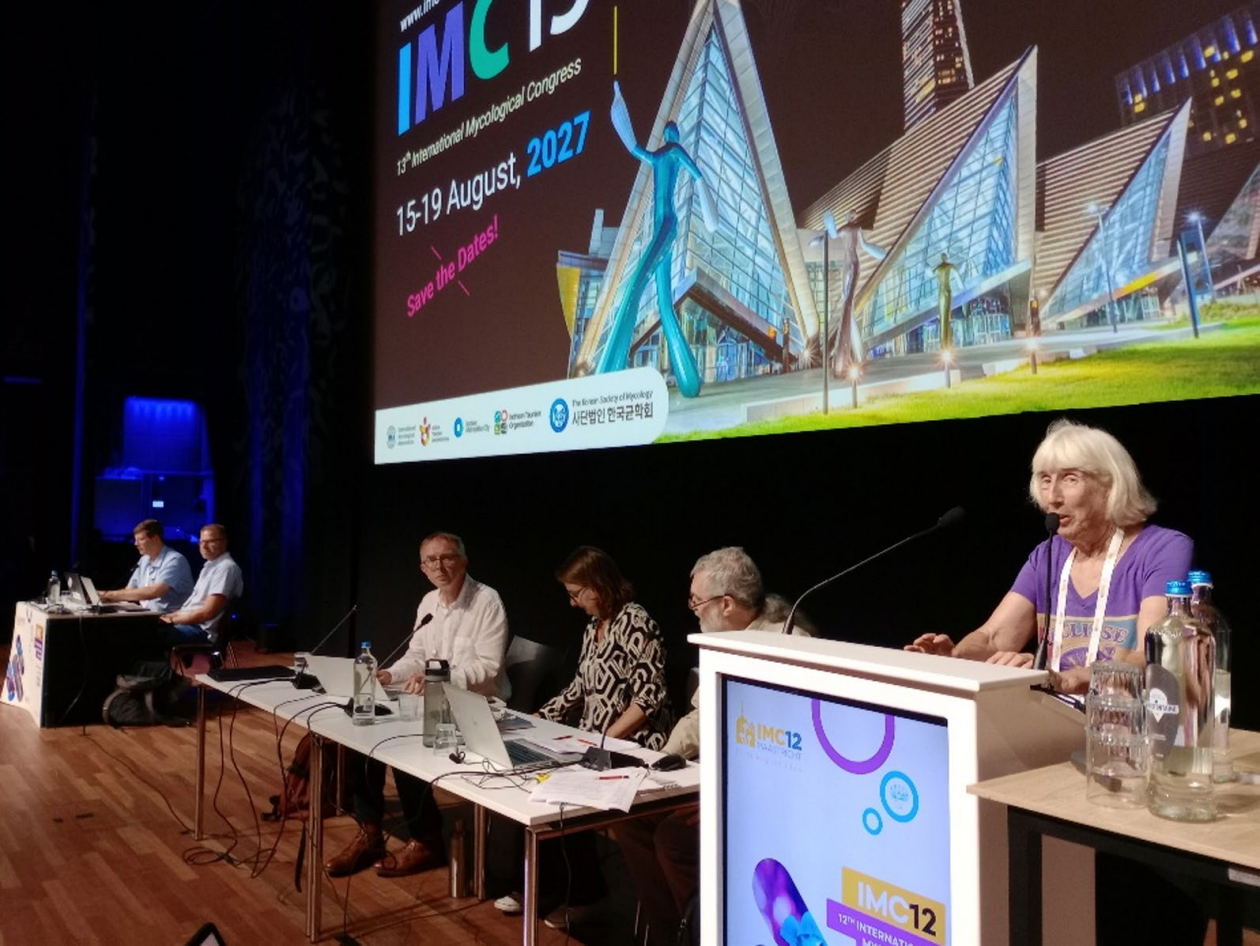
Members of the Fungal Nomenclature Bureau, IMC12 (*left to right*): Ewald Groenewald (Reporter), Jos Houbraken (Reporter), Nicholas Turland (Rapporteur-général XXI IBC, advisor), Konstanze Bensch (Deputy Secretary), Tom May (Secretary) and Amy Rossman (Chair)

Division III of the ICNafp (Provisions 4 and 8) sets out officers of the Fungal Nomenclature Bureau (FNB) and the procedures for their appointment. The FNB of an International Mycological Congress is responsible for running the FNS and the pre-Congress *Guiding Vote*. The **Fungal Nomenclature Bureau** of IMC12 (Fig. 2) comprised Amy Rossman (Corvallis, USA, Chair), Tom W. May (Melbourne, Australia, Secretary), Konstanze Bensch (Utrecht, The Netherlands/Munich, Germany, Deputy Secretary), Ewald (J.Z.) Groenewald and Jos Houbraken (Utrecht, The Netherlands, Recorders), and five Deputy Chairs. The Deputy Chairs, as appointed by the FNS, were David L. Hawksworth (Kew and London, UK, Deputy Chair Emeritus), M. Catherine Aime (West Lafayette, USA), Lei Cai (Beijing, China), Pedro Crous (Utrecht, The Netherlands) and Irina Druzhinina (Kew, UK). In addition, Nicholas J. Turland (Berlin, Germany), Rapporteur-général for the XXI International Botanical Congress, attended as a non-voting advisor to the FNS on the invitation of the International Mycological Association.

The **Nominating Committee** of the International Mycological Congress was proposed by the Chair of the FNS, in consultation with the other members of the FNB, and was accepted by the FNS. Composition of the Nominating Committee was as follows: Tatiana Gibertoni (Pernambuco, Brazil, Chair), Lei Cai (Beijing, China), David L. Hawksworth (Kew and London, UK), Meike Piepenbring (Frankfurt am Main, Germany) and Irina Druzhinina (Kew, UK). The function of the Nominating Committee was to prepare a list of candidates to serve on the permanent Nomenclature Committee for Fungi (NCF), in consultation with the current Secretary of that Committee; prepare a list of candidates for the Editorial Committee for Fungi; and propose the Secretary of the Fungal Nomenclature Bureau for the next International Mycological Congress.

Procedures for the FNS are set out in Division III of the ICNafp. These procedures closely follow those of the Nomenclature Section of an IBC, except that there are no institutional votes.

The FNS accepted the Shenzhen ICNafp (Turland et al. 2018) as the basis for its deliberations, noting that *Chapter F* has been amended at IMC11 and published as the San Juan *Chapter F* (May et al. 2019).

It has been traditional at Nomenclature Sections of IBCs to deal with proposals in the sequence in which articles and other material to be amended (or added) appear in the ICNafp. However, due to the relatively small number of proposals this practice was not followed in Maastricht. Rather, in order to allow for discussion of the two main proposals about DNA sequences as types for fungi at the lunchtime session and to group some proposals dealing with similar matter together, proposals were dealt with in the following order: F-002, F-003, F-004, F-001, F-008, F-005, F-006, F-007, F-010 and F-011, noting that five of the 15 proposals were withdrawn prior to the Session.

Proceedings of the IMC11 and IMC12 FNS will be published in full in due course, based on transcripts of audio recordings of the Sessions. This follows the tradition of publishing the proceedings of Nomenclature Sections of IBCs, the most recent being that of the Shenzhen IBC (Lindon et al. 2020).

Each person present at the FNS (except for the Rapporteur-général) had one vote. Members of the FNB were eligible to vote, but, in order not to overly influence the voting, did not necessarily choose to vote on all matters. Voting in the FNS was initially by show of hands, but, when the required majority for voting was not clear, a “card vote” was held, where persons present cast their votes by writing “yes” or “no” on slips of paper, which were counted by tellers appointed by the Chair.

The FNS accepted the candidates put forward by the Nominating Committee for the position of Secretary of the Fungal Nomenclature Bureau and the members of Nomenclature Committee for Fungi for the period 2024–2027 as follows:

**Secretary of the Fungal Nomenclature Bureau for the XIII International Mycological Congress, Incheon, South Korea, 2027**.—Tom W. May (Australia).

**Nomenclature Committee for Fungi**.—Konstanze Bensch (The Netherlands/Germany, Chair), Tom W. May (Australia, Co-Secretary), James C. Lendemer (USA, Co-Secretary), Mounes Bakhshi (UK), Mika Bendiksby (Norway), Marcela Cáceres (Brazil), Cecile Gueidan (Australia), Teresa Iturriaga (Venezuela/USA), Paul M. Kirk (UK), Roland Kirschner (Taiwan), James K. Mitchell (USA), Andrew M. Minnis (USA), Luis A. Parra Sánchez (Spain), Scott A. Redhead (Canada), Svengunnar Ryman (Sweden), Marco Thines (Germany), Ke Wang (China), and Zhuliang Yang (China).

## PROPOSALS FROM THE FLOOR

New proposals (“Proposals from the floor”), which had not been included in the *Synopsis of proposals*, were accepted by the Secretaries up to the commencement of the FNS (10:30 h on Thursday 15 August 2024). Proposals from the floor (Table 1) were numbered by continuing the sequence used in the *Synopsis of proposals*. All but one of the eight proposals from the floor had appeared in a publication prior to the FNS (Yurkov et al. 2024), while the other (F-008) was provided directly to the Secretaries. For the set of proposals arising from Yurkov et al. (2024), discussions were held between the proposers and the Secretaries prior to the FNS, resulting in the proposers agreeing to withdraw all but two of the set of proposals. These two proposals as presented to the FNS (Prop. F-010 and F-011) were modified from those originally published by Yurkov et al. (2024).

**Table 1:** Actions on nomenclature proposals considered by the Fungal Nomenclature Session of IMC12, including “proposals from the floor”

Prop. no.	Proposal	% “No” in *guiding vote*	Congress action
*Proposals included in Synopsis of proposals* (May and Bensch 2024)			
F-001	Enable same epithet to be retained for different morphs of same fungus	27	Rejected
F-002	Clarify that a proposal to conserve a name with a conserved type does not require citation of a typification identifier	13	**Accepted**
F-003	Remove the listing of synonyms from entries for protected names in the appendices to the *Code* and clarify the processes of protection under Art. F.2.1	19	**Accepted**
F-004	Clarify the processes of rejection under Art. F.7.1	5	**Accepted**
F-005	Allow the naming of fungi from DNA sequences as types	80	Rejected *Guiding Vote*; not reintroduced
F-006	Allow genomic sequences to serve as types of names of organisms treated as fungi	74	Special-purpose Committee
F-007	Add a recommendation on the designation of fungal organisms only known from DNA sequence data	69	Rejected
*Proposals "from the floor" *[proposed by]			
F-008	In Art F.2.1, clarify that an earlier homonym of a sanctioned name remains unavailable if the sanctioned name is rejected outright [Minnis and May, provided directly to the Secretaries]	n/a	**Accepted**
F-009	The holotype strain and any isotype strains must be registered [Yurkov et al. (2024)]	n/a	Withdrawn
F-010	A modification of Rec. 8B.1, specific to fungi, to add that culture collections or biological resource centres should be “public” [Yurkov et al. (2024)]	n/a	**Accepted after amendment—**Note 1
F-011	Add a new recommendation that when original type cultures are lost, should neotypify on progeny of ex-type cultures [Yurkov et al. (2024)]	n/a	**Accepted after amendment—**Note 2
F-012	Any type of writing that explicitly indicates in a non-contradicting way that a single culture is the holotype is to be considered a valid typification [Yurkov et al. (2024)]	n/a	Withdrawn
F-013	Acronyms of culture collections (when not explicitly explained in a description) can be determined according to data sources managed by the WFCC-MIRCEN World Data Center for Microorganisms. Any other culture identifiers are to be interpreted as strain designations [Yurkov et al. (2024)]	n/a	Withdrawn
F-014	Recommended format for typification of names of fungi based on cultures [Yurkov et al. (2024)]	n/a	Withdrawn [convert to examples, refer to Fungal Editorial Committee]
F-015	Before 1 Jan 2019, when “metabolically inactive state” is not clearly stated, treat as correctable error not preventing valid publication, provided there is evidence that a type was preserved in a metabolically inactive state by the specified culture collection prior to publication [Yurkov et al. (2024)]	n/a	Withdrawn

Note 1. The proposal, which as presented had been modified from that originally presented in Yurkov et al. (2024), was further amended to read: “When applying Rec. 8B.1 to the name of a fungus, the collections should be public.”

Note 2. The proposal, which as presented had been modified from that originally presented in Yurkov et al. (2024), was further amended to read: “When the name of a fungus was based on a type that is a living culture in a metabolically inactive state and a neotype is chosen (Art. 9.8), that neotype should be the oldest progeny of an ex-type culture preserved metabolically inactive.”

## ACTIONS ON PROPOSALS

There were 15 proposals. Actions of the FNS on these are summarized in Table 1. Five proposals were withdrawn, one of which was referred to the Editorial Committee for Fungi, one was sent to a Special-purpose Committee that was established at the FNS (see below), one was rejected in the *Guiding Vote* (and not reintroduced), two were rejected, and six were accepted (two after amendment). A card vote was held on Prop. F-007 because the visual counting of hands by three members of the FNB yielded a 60.09% “yes” vote, extremely close to the required 60%, but with discrepancies between the three counts. The card vote yielded a 51.76% “yes” vote, hence the proposal was not accepted. Successful proposals are indicated in Table 1 and a brief precis of each is provided in Box 1.

**Box 1:** Key outcomes of the IMC12 Fungal Nomenclature Session

**ICNafp** *amendments:*	
Clarified that a proposal to conserve a name with a conserved type does not require citation of a typification identifier	
Clarified the processes of protection under Art. F.2.1 and rejection under Art. F.7.1 as well as removed the need to list synonyms from entries for protected names in the appendices to the *Code*	
Clarified that an earlier homonym of a sanctioned name remains unavailable if the sanctioned name is rejected outright	
Introduced a modification of Rec. 8B.1, specific to fungi, that culture collections or biological resource centres should be “public”	
Added a new recommendation that, when original type cultures are lost, neotypification should utilize the progeny of ex-type cultures	
***Special-purpose Committee to report to IMC13 on:***	
Genomes as types for fungi	
***Fungal Editorial Committee:***	
To add example/s demonstrating the recommended format for typification of names of fungi based on cultures	
***Chapter F of the ICNafp and the Madrid* Code**	
The revised version of *Chapter F* will appear as an integral part of the Madrid *Code*. This edition of the *Code* will be published in 2025 and also made available online via the website of the International Association for Plant Taxonomy (IAPT). There will be no separate “Maastricht *Chapter F*”	

## PERMANENT NOMENCLATURE COMMITTEE REPORT

The Secretary of the Nomenclature Committee for Fungi, Tom W. May, presented the following report:

“Over the last 6 years, the NCF has finalized decisions on proposals to conserve or reject 53 names and referred a further three proposals to the General Committee without a recommendation. Nine proposals related to names of fungi were withdrawn or are no longer relevant (due to changes to the wording of the *Code*). In addition, the NCF has examined and approved five lists from international working groups, consisting of 50 names proposed for protection. The matters finalized deal with 115 names of taxa and the suppression of three works. Finalized matters have been covered by three reports, published in 2023 and 2024. A further report on the remaining 30 resolved matters is in preparation.

The NCF also approved the composition of two Special-purpose Committees to report to IMC12 and was consulted about the appointment of officers of the Fungal Nomenclature Bureaux of IMC11 and IMC12.

The Secretary apologizes for the long delay in processing some proposals and acknowledges that there remains a backlog of 34 proposals still under consideration.

When “fungi governance” passed to the IMA, a number of new functions had to be put in place including the Fungal Nomenclature Bureau and the ad hoc (now formalized) Editorial Committee for Fungi. High overlap between officers of the NCF and these other bodies means that the Secretary of the NCF, in particular, has a particularly high workload. Steps in place to reduce this are under consideration including splitting the role of Secretary and introducing a mentoring programme so that younger mycologists can receive exposure to and training in the work of the NCF. Of the potential joint Secretaries, only one Secretary would take up the mandated *ex officio* position on the General Committee.

The current composition of the NCF was approved by IMC11, in San Juan, Puerto Rico in 2018, with 21 members. Membership has declined to 16, with the death of our highly esteemed former Secretary Lorelei Norvell, the retirement of Yi-Jian Yao, Andrea Romero, Z. Wilhelm de Beer, and the resignation of Lorenzo Lombard. Retiring at IMC12 are Andre Aptroot, Dagmar Triebel, and Shaun Pennycook. All NCF members past and present are thanked for their collegial discussions.”

As mentioned in the report from the Secretary of the NCF, since IMC11 in 2018, three reports have been published: Reports 22, 23, and 24 (May and Lendemer 2023; May 2024a, 2024b). It should be noted that the recommendations of the Nomenclature Committee for Fungi are next considered by the General Committee, whose decisions are ultimately accepted or not by the Nomenclature Section of an International Botanical Congress. In practice, it is rare for the General Committee not to accept a recommendation of the NCF. The Reports of the General Committee that dealt with the recommendations of the NCF since the last IBC (as included in NCF Reports 22, 23 and 24), were unanimously accepted at the Nomenclature Section of IBCXX (Turland et al. 2024).

## SPECIAL-PURPOSE COMMITTEES

One of the acceptable actions during the Fungal Nomenclature Session is to establish a Special-purpose Committee (SPC) [ICNafp Div. III, Prov. 8.5.(d)]. During discussion of proposal F-006 “to allow genomic sequences to serve as types of names of organisms treated as fungi”, a Special-purpose Committee was proposed and authorized by the FNS, to report to IMC13 in 2027. The title of this SPC was originally proposed as “Genomes as Types”. Here, it is amended to “Genomes as Types for Fungi” to be specific as to its mandate in the context of other SPCs that may be in operation. There was some discussion during the FNS as to the appropriateness of the terms “Genomes” and “Genomic sequences” to cover the remit of the SPC, such as in relation to the treatment of Digital Sequence Information in the Kunming-Montreal Global Biodiversity Framework. The SPC will be instructed to take into account any potential issues around terminology for “genomes”.

Membership of Special-purpose Committees is approved by the NCF in consultation with the General Committee [Div. III, Prov. 8.5.(d)]. A call was made during the FNS for expressions of interest for the new SPC on Genomes as Types for Fungi and a similar call has been distributed to the members of the NCF and to those who served on the previous Special-purpose Committee on DNA Sequences as Types for Fungi. The composition of the SPC on Genomes as Types for Fungi is expected to be finalized by early 2025 and the members will be listed in the next report of the NCF.

There is already a Special-purpose Committee on Typeless Names and DNA Sequences as Types, established at the Madrid IBC, to report to the 2029 IBC, dealing with all organisms covered by the ICNafp and dealing with all DNA sequences, not just genomes. Liaison between these two SPCs, one reporting to the IMC and the other to the IBC but with overlapping mandates, will be advisable.

## RESOLUTION AND THE NEW WORDING OF ICNafp *CHAPTER F*

As its final item of business, the FNS accepted a motion from the Secretary that the Chair and the Secretaries be instructed to present to the President of the International Mycological Association (IMA) a resolution on behalf of the Session to the effect that the decisions and appointments of the FNS be approved. At the closing ceremony (plenary session) to the Congress, which began at 17:00 local time on Thursday 15 August 2024, the following resolution was accepted:

“The XII International Mycological Congress resolves that the decisions of its Fungal Nomenclature Session with respect to Chapter F of the International Code of Nomenclature for algae, fungi, and plants, as well as the appointment of the Secretary of the Fungal Nomenclature Bureau for IMC13, and officers and members of the Nomenclature Committee for Fungi and the Fungal Editorial Committee, made by that Nomenclature Session during its meeting on 15 August 2024, be accepted, noting with interest the formation of a Special-purpose Committee on Genomes as Types.”

The wording of *Chapter F* of the *Code* will be amended in accordance with this resolution, by the Editorial Committee for Fungi, as a recommendation to the full Editorial Committee of the ICNafp, who will meet in late November 2024 to prepare the Madrid *Code*. Due to the close timing of the IBC and IMC in 2024, rather than prepare a separate *Chapter F*, the amendments to *Chapter F* will instead be incorporated directly into the Madrid *Code*, which is expected to appear in early 2025, in both print and on-line versions. Thus, there will be no separate publication of a “Maastricht *Chapter F*”.

Amendments to the ICNafp became effective **immediately** upon acceptance of this resolution; they are not dependent upon publication of the present report nor upon publication of the revised version of *Chapter F* in the Madrid *Code*. New and amended rules are retroactive to the starting-point date for the nomenclature of fungi, 1 May 1753 (Art. F.1 and 13.1(f)), unless expressly limited. However, no decisions were made in Maastricht that were date limited.

## RECOMMENDATIONS FOR FUTURE FUNGAL NOMENCLATURE SESSIONS

The format of the Nomenclature Section of the International Botanical Congress, as a 5-day session the week prior to the IBC, is well-established. In contrast, the two Fungal Nomenclature Sessions, at IMC11 in San Juan and IMC12 in Maastricht, have operated differently, and there remain some issues to fine tune. In San Juan, the FNS was held on the middle day of a five-day Congress and was not concurrent with any of the formal business of the Congress. In Maastricht, the FNS was held on the final day of a four-day Congress, and the morning and afternoon sessions were concurrent with the Congress symposia. Concurrent symposia meant that scheduling the order of consideration of proposals was challenging, due to proposers or those with a particular interest in proposals having other commitments, such as speaking or chairing concurrent sessions. The need to provide access to all delegates to debate and vote on the significant proposals about DNA sequences as types for fungi meant that, at late notice, an additional session had to be organised during the lunch break, which was not ideal. **We recommend that the FNS is not concurrent with Congress symposia at IMCs**. Whether the FNS is scheduled within or before the Congress is a matter for further consideration. The plus side of holding the FNS during the Congress, is that a greater number of Congress delegates are likely to be able to attend (in comparison to holding the Session prior to the Congress, either on the weekend, or the Friday prior). The negative side is that conflicts with parallel sessions are difficult to avoid. Ultimately, the length of time devoted to the FNS depends on the number of proposals submitted, and this is not known until around six months out from the Congress.

The audio-visual set up of the room in which the FNS is held contributes to its efficient running. Essential facilities are hand-held microphones as used both in San Juan and Maastricht, which allows speakers from the floor to be clearly heard by the rest of the audience. Only one screen was available in Maastricht during the IMC12 FNS. It is ideal to have two screens, so that the wording of the *Code* and the wording of proposals can be presented, side by side. Adding line numbers to the text of the proposals as presented on the screen would assist in locating the position of amendments. The Recorders were seated on the other side of the stage from the rest of the Fungal Nomenclature Bureau, which at times made communication difficult, such as when consulting on the exact wording of amendments. It would work better for the Recorders to be next to the Secretaries. The high stage and the large auditorium in Maastricht meant that members of the FNB found it difficult to see who was speaking and also to hear clearly, as the loudspeakers were in front of the Fungal Nomenclature Bureau and faced away from the stage. Testing of the set-up beforehand is advisable at future IMCs to resolve such issues. If a similarly scaled stage setup is used in the future, the addition of floor-monitors (loudspeakers facing the stage) is strongly advised to improve the sound quality on the stage. An additional recommendation is to have the session recorded by the AV personnel through the mixing table if possible, as this allows for a high-quality sound recording for transcription purposes compared to using a traditional handheld recorder.

The size of the auditorium has to be large enough to accommodate the likely number of attendees. Without registration, it was difficult to predict the number of attendees. **For the FNS at IMC13, specific pre-registration is advisable**, but with flexibility for late registrations.

## FUTURE PROPOSALS

IMC13 will be held in Incheon, South Korea, 15–19 August 2027. The procedures and timetable for proposals to amend *Chapter F* to be considered at the FNS of IMC13 will be published early in 2025 in *IMA Fungus*. The procedures will be largely the same as those in place for IMC12 (May 2020) and the deadline for submission of proposals will be 31 December 2026. For proposals to amend *Chapter F* that are major changes, those making proposals are advised to publish proposals even earlier, ideally by the end of 2025. Early publication of proposals will facilitate full and informed discussion across the mycological community and provide sufficient time to foster development of the consensus and wide community support that contributes to the successful adoption of proposals to amend the *Code* that are significant changes.

## WESTERDIJK SPRING SYMPOSIUM ON DNA AS TYPE

Given the ongoing interest in the topic of DNA sequences as types for fungi, the Westerdijk Fungal Biodiversity Institute will hold their next Spring Symposium on the topic of “DNA as Type” on 7–8 April 2025, in Utrecht, The Netherlands (https://wi.knaw.nl/news/details/129853). This meeting will be an important opportunity for the Special-purpose Committee on Genomes as Types for Fungi and the wider mycological community to discuss issues and work towards a consensus wording of proposals to amend *Chapter F* in relation to this topic, should such proposals be considered desirable.
